# Bilateral olfactory aplasia: Uncommon cause of congenital anosmia

**DOI:** 10.1002/ccr3.2218

**Published:** 2019-06-07

**Authors:** Ashraf Nabeel Mahmood, Osama Abulaban, Arshad Janjua

**Affiliations:** ^1^ Heartlands Hospital, University Hospitals Birmingham NHS Foundation Trust Birmingham UK

**Keywords:** anosmia, aplasia, congenital, olfactory

## Abstract

Olfactory aplasia is an uncommon cause of anosmia, and it should be suspected in young patients with lifelong decreased sense of smell. The aim of this article was to remind readers about this condition that need to be part of the differential diagnosis in similar clinical scenario, to avoid unnecessary investigation and overtreatment for these patients.

A 10‐year‐old healthy girl presented with nasal congestion and a lifelong anosmia. She had adenotonsillectomy at the age of 5 years. Examination of the nose showed no structural deformities or obstructive lesions. Neurological examination and cognitive development assessment were normal for her age. Blood tests including immunoglobulin E (IgE) level and radioallergosorbent test (RAST) were normal. Magnetic resonance imaging (MRI) scan performed to rule out underlying anterior cranial fossa or intranasal pathologies (Figure 1A‐C).

**Figure 1:**
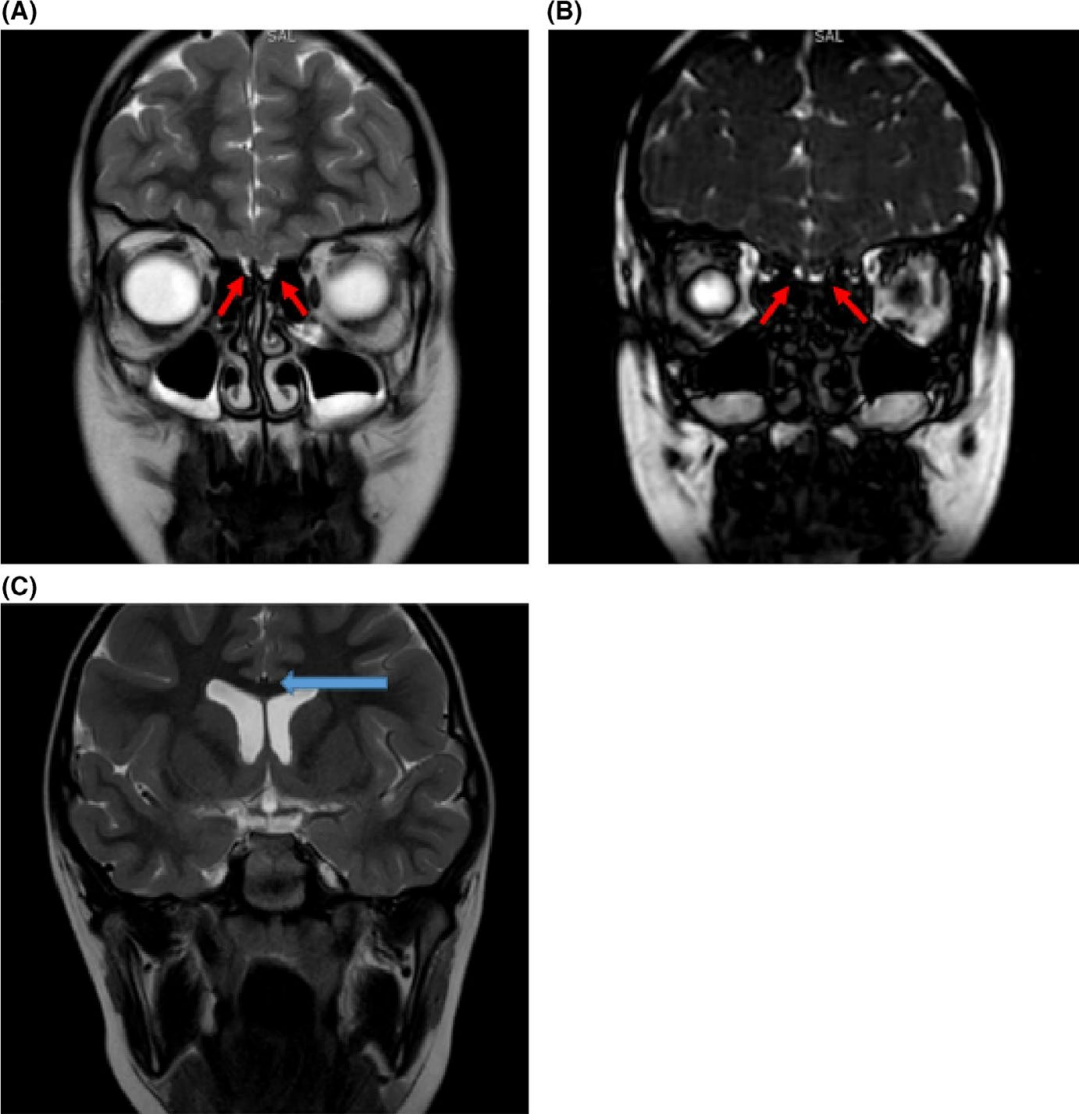
A, (T2‐weighted MRI), B, (high‐resolution T2‐weighted MRI) images, showing bilateral absence (aplasia) of the olfactory nerve with preserved olfactory sulci (Red arrows). C, (T2‐weighted MRI) showing preserved septum pellucidum and corpus callosum with no dysgeneses of posterior fossa structures (Blue arrow)

## 1 WHAT IS THE DIAGNOSIS?

### 1.1 Answer: Bilateral olfactory aplasia

Primary anosmia as a result of olfactory aplasia is uncommon pathology with the incidence of 700 per 1 000 000 births.^1^ It can present as part of other syndromes or, less commonly, as isolated malformation. Radiological investigations should be considered in young age patients with lifelong anosmia but no suggestive causes of their symptoms by clinical history or nasal examination, to rule out intracranial and olfactory tract abnormalities. MRI scan is the most reliable imaging modality in these cases. Also, olfaction can be tested by using nonirritating odorous substances and smell tests like University of Pennsylvania Smell Test (UPSIT), Sniff test, or combined olfactory test.

Even though there is no curative treatment for anosmia caused by olfactory aplasia, proper counseling is essential.^2^ Patient needs to be counseled about daily activities and precautions like fitting smoke and gas detectors, avoid using gas cookers, and the importance of checking the expiry date of the food products.

## CONFLICT OF INTEREST

None declared.

## AUTHOR CONTRIBUTION

AM: reviewed the literature, designed the case report, prepared and the manuscript as first author. OA: performed, reviewed and prepared the MRI scan images. AJ: assessed the patient, edited, supervised and critically reviewed the manuscript.
